# Estudio sobre el valor incremental que supone el informe del sedimento en orina en medicina de urgencias mediante el sistema Sysmex^®^ Serie-UN

**DOI:** 10.1515/almed-2024-0181

**Published:** 2024-11-26

**Authors:** Marco Tosi, Davide Negrini, Giovanni Celegon, Martina Montagnana, Giuseppe Lippi

**Affiliations:** Sección de Bioquímica Clínica y Facultad de Medicina, Universidad de Verona, Verona, Italia

**Keywords:** urianálisis, análisis de sedimentos urinarios, medicina de urgencias, técnicas de laboratorio clínico

## Abstract

**Objetivos:**

El análisis de orina es ampliamente utilizado y frecuentemente solicitado desde los servicios de urgencias para evaluar la presencia de hipovolemia, infecciones del tracto urinario, diabetes, cetoacidosis y hematuria. Nuestro objetivo era evaluar el impacto de informar el sedimento urinario en muestras enviadas desde el servicio de urgencias utilizando el analizador Sysmex^®^ Serie-UN.

**Métodos:**

Evaluamos los análisis de orina solicitados por el servicio de urgencias durante un periodo de tres meses e investigamos la interferencia del recuento de hematíes, comparamos las tiras reactivas de esterasa leucocitaria con el recuento de leucocitos citofluorimétrico y los nitritos con el recuento bacteriano citofluorimétrico. A continuación, examinamos las imágenes con el microscopio digital para identificar otros posibles elementos o patologías de interés.

**Resultados:**

Recopilamos 532 casos, en 354 de los cuales solo se disponía de análisis bioquímico y citofluorimetríco, y en 178 se disponía además de análisis por microscopía digital. El recuento automatizado de eritrocitos mostró una tasa de error del 7 %, principalmente falsos positivos. La esterasa leucocitaria tuvo una sensibilidad del 88,22 % y una especificidad del 88,84 % en el límite inferior, mientras que los nitritos tuvieron una sensibilidad del 41,06 % y una especificidad del 99,38 %. Se detectaron elementos patológicos en 126 muestras mediante microscopía digital, de los cuales 70 presentaban cilindros, 36 cristales y 7 contenían células de elevado valor patológico.

**Conclusiones:**

La evaluación de los sedimentos en orina por parte de especialistas capacitados puede aportar información potencialmente relevante incluso en casos urgentes, donde la fase preanalítica debe ser siempre tenida en cuenta.

## Introducción

El análisis de orina es una prueba cuyo uso está muy extendido, ya que proporciona información clínica relevante, la recogida de muestras es relativamente sencilla, se puede realizar de forma segura y precisa en prácticamente cualquier laboratorio y presenta una buena relación coste-beneficio. Junto con la determinación de creatinina y el cálculo de tasa de filtración glomerular estimada (FGe), el análisis de orina es la primera prueba que se realiza para el diagnóstico de enfermedad renal y lesión o disfunción del tracto urinario [1, 2].

El análisis de orina suele incluir algunos o la totalidad de los siguientes aspectos: examen físico (color y aspecto), determinación de la concentración de orina (densidad relativa u osmolaridad), determinaciones químicas (proteínas, glucosa, cetonas, hemoglobina, esterasa leucocitaria, nitritos, pH y otras pruebas), examen del sedimento urinario (utilizando analizadores automáticos para contar los componentes corpusculares y/o análisis microscópicos para un análisis más detallado de los diversos elementos detectados) [3, 4].

En la práctica clínica, el urianálisis se suele realizar ante la sospecha de infección del tracto urinario, para evaluación inicial o seguimiento de la enfermedad renal, de enfermedades no infecciosas del tracto urinario (primarias o causadas por patologías sistémicas, como enfermedades reumatológicas, hipertensión, otras patologías del embarazo o los efectos secundarios de tratamientos farmacológicos) y en el caso de formación recurrente de litiasis renal [3, 5].

El análisis de orina también se suele solicitar en los servicios de urgencias, principalmente como herramienta de detección para evaluar estados de hidratación e hipovolemia (p.ej. evaluando la concentración de la orina) [6], ante la sospecha de infecciones del tracto urinario (debido al elevado valor predictivo de la esterasa leucocitaria y la positividad para los nitritos) [7], en casos de diabetes y cetoacidosis diabética (para evaluar la concentración de glucosa y cetonas) y para evaluar la pérdida de sangre por el tracto urinario (hematuria). Así mismo, con los análisis químicos de orina se pueden evaluar otras patologías, ya que proporcionan información útil a la hora de establecer un diagnóstico diferencial rápido y preciso [8].

Concretamente, los análisis de orina siempre se deberían solicitar sobre la base de una sospecha clínica concreta, ya que las solicitudes inadecuadas pueden complicar la evaluación de la prueba [9]. Aunque las pruebas físicas y químicas se suelen realizar en la mayoría de los laboratorios clínicos, la evaluación del sedimento urinario en el contexto de las urgencias se suele realizar únicamente si se solicita específicamente. Por este motivo, se puede perder información potencialmente relevante que las tiras reactivas no ofrecen.

El objetivo del presente estudio no era evaluar el rendimiento del sistema Sysmex^®^ Serie-UN: (sobradamente documentado en la literatura), sino evaluar el posible impacto (en cuanto al número de muestras que el analista debe comprobar, modificar o comentar) de incluir también el informe del sedimento urinario en las muestras remitidas por el servicio de urgencias (en el que actualmente solo se comunican los resultados de la tira reactiva). Otro objetivo de este estudio era analizar la concordancia de algunos parámetros útiles entre los resultados de la tira reactiva y la citofluorimetría (en los que se basan la mayoría de las reglas de verificación cruzada) y los resultados del sistema de urianálisis Sysmex^®^ Serie-UN. Este sistema está compuesto por un analizador químico, una plataforma de citofluorimetría (para detectar sedimentos urinarios y reducir el número de muestras que precisan evaluación microscópica) y un microscopio automatizado.

## Materiales y métodos

### Recopilación de datos

En este estudio observacional retrospectivo, evaluamos todas las peticiones de análisis de orina enviadas por el servicio de urgencias del Hospital Universitario de Verona durante tres meses (entre febrero y abril de 2023). Para cada petición, la orina fue analizada empleando el sistema modular de Sysmex^®^ Serie-UN (Sysmex Corporation, Kobe, Japón), compuesto por un Sysmex UC-3500 (analizador químico automático) que utiliza tiras reactivas MediTape UC-11A, el analizador Sysmex UF-4000 (método citofluorimetríco) y el sistema Sysmex UD-10 (microscopio digital). Las muestras de orina son enviadas al laboratorio por el servicio de urgencias mediante tubos neumáticos para su posterior análisis en los siguientes 30 minutos.

Todos los análisis se realizaron con los analizadores UC-3500 y UF-4000. Todas las muestras, así como sus parámetros específicos o una combinación de los mismos, fueron examinadas con el microscopio digital UD-10 con el fin de obtener imágenes de campo brillante del sedimento urinario, para su evaluación morfológica. Dos profesionales de laboratorio formados en análisis de orina revisaron los datos e imágenes de todas las muestras y consensuaron la interpretación de los resultados.

Los datos obtenidos para el presente estudio incluyeron la totalidad de los resultados obtenidos mediante instrumentación, extraídos con el programa DMS-ANUR (Dasit Group, Milan, Italia), incluyendo cualquier corrección realizada previamente al informe de los resultados, y los comentarios añadidos tras la evaluación morfológica del sedimento urinario, en su caso.

En la Tabla Suplementaria S.1. se muestran las normas adoptadas por nuestro laboratorio sobre el *middleware* Sysmex UN (DMS-ANUR) para la puesta en marcha de la microscopía digital automatizada. La lista de parámetros incluidos en el estudio, los intervalos de referencia y el estado actual de inclusión de cada parámetro en los informes se muestran en la Tabla Suplementaria S.2.

En el informe del análisis de sedimento urinario se incluía el recuento eritrocitario (si >15/µL), leucocitos (si >20/µL), bacterias (si >1,000/µL), y células epiteliales escamosas (si >20/µL), proporcionados por el analizador. El recuento de elementos de las demás categorías no se incluye en el informe final, ya que únicamente es útil como alerta para la evaluación de imágenes digitales. De este modo, estos otros elementos de posible interés clínico se incluyen como comentarios descriptivos con evaluaciones semi-cuantitativas (pocos, algunos, numerosos, muy abundantes).

### Interferencia entre el recuento eritrocitario en el canal SF_RBC/X’TAL

El diagrama de dispersión eritrocitaria es susceptible de sufrir interferencias [10, 11], que pueden manifestarse como una distribución anormal de los eritrocitos, mostrando curvas de dispersión o “en gancho”. La dispersión SF_RBC/X’TAL construye un gráfico entre la intensidad de la luz despolarizada con dispersión lateral y la intensidad de la luz con dispersión frontal, para distinguir los glóbulos rojos (ubicados en una zona de baja intensidad de la luz con dispersión lateral) de los cristales (que despolarizan la luz), pero a veces puede verse interferido por cristales de baja despolarización u otras partículas. También se examinó el número de muestras en las que el número de eritrocitos/μL proporcionado por el instrumento cambió debido a una interferencia en el recuento.

### Comparación de la tira reactiva de esterasa leucocitaria con el recuento citofluorimétrico de leucocitos

Dada la prevalencia de los falsos negativos, se evaluó el valor añadido del recuento citofluorimétrico de leucocitos con respecto a la utilización de tiras reactivas en la evaluación de la esterasa leucocitaria [12]. La sensibilidad y especifidad se evaluaron a dos niveles: el primero al límite discriminante de positividad (1+ o más) para la tira reactiva y 20 elementos/µL para el recuento de leucocitos mediante citofluorimetría; el segundo a valores más altos de positividad de la tira reactiva de al menos 2+ (parámetro del fabricante para positividad de tira reactiva de 2+) y 75 elementos/µL para los leucocitos en citofluorimetría.

### Comparación de nitritos en tira reactiva con recuentos bacterianos citofluorimétricos

Se comparó el valor incremental del recuento bacteriano citofluorimétrico, con los nitritos obtenidos mediante tira reactiva, teniendo en cuenta la frecuente presencia de falsos negativos ya que, tal como hemos mencionado anteriormente, no todas las bacterias producen nitritos. Se evaluaron la sensibilidad y especificidad al nivel discriminante de positividad, esto es, positivo para tira reactiva, y 1,000/μl para bacterias en citofluorimetría (umbral definido en el laboratorio para “poco frecuente”).

### Otros elementos o patologías de interés (microscopía digital)

Las muestras se analizaron mediante microscopía digital para identificar otros posibles elementos o patologías de interés a las categorías de cuantificación automática (eritrocitos, leucocitos, bacterias, células epiteliales escamosas).

## Resultados

### Recopilación de datos

Durante el periodo de observación de tres meses, se revisaron 532 casos de pacientes asistidos en el Servicio de Urgencias, correspondientes a 265 hombres y 267 mujeres, con una media de edad de 64.02 (desviación estándar 22.54) y una mediana de edad de 69 años (percentiles del 25 al 75: 49–83 años). Un total de 354 de las 532 muestras fueron analizadas únicamente con los sistemas UC-3500 y UF-4000, mientras que los 178 restantes también se analizaron mediante microscopía digital con un microscopio UD-10. En 126 de las 178 peticiones de pruebas analíticas evaluadas con imágenes de UD-10, la presencia de elementos formes que no fueran eritrocitos, leucocitos, bacterias y células escamosas se comunicó en un comentario específico.

### Interferencia en el recuento eritrocitario en el canal SF_RBC/X’TAL

Hubo 37 errores (6,95 % del total) en el recuento automático de eritrocitos: 26 falsos positivos (4,89 % del total de peticiones; 70,27 % de errores), debido a interferencias, en las que los eritrocitos fueron detectados por los analizadores a pesar de su ausencia; 11 errores de sobreestimación (2,06 % de todas las solicitudes; 29,73 % de los errores) fueron causados ​​por una distribución anormal de los eritrocitos en el diagrama de dispersión.

### Comparación de la tira reactiva de esterasa leucocitaria con el recuento citofluorimétrico de leucocitos

En el punto discriminante de positividad (1+ para esterasa leucocitaria y >20/μL para el recuento citofluorométrico), la sensibilidad fue del 88,22 %, mientras que la especificidad fue del 88,84 %. La sensibilidad fue del 91,18 % y la especificidad del 94,17 % en el punto discriminate más alto para la presencia de leucocitos (2+ para esterasa leucocitaria y 75/μL para el recuento citofluorimétrico).

### Comparación de nitritos en tira reactiva con recuentos bacterianos citofluorimétricos

La sensibilidad en el punto de corte o discriminante fue del 41,06 %, con una especificidad del 99,38 %.

### Otros elementos o patologías de interés (microscopía digital)

Se detectaron elementos o patologías de interés en 126 de las 178 muestras examinadas (71 %) mediante microscopía digital: 70 muestras presentaban cilindros (73 cilindros hialinos y 27 cilindros más patológicos, como hialino-granulosos- y granulosos, habiéndose detectado la presencia de cilindros céreos en un caso).

En cuanto a la presencia de células epiteliales no escamosas (39 muestras), 32 muestras contenían células de escaso valor patológico (células de transición superficiales), mientras que 7 presentaban células de mayor valor patológico (transición profunda, tracto superior, tubular renal). Se hallaron levaduras en 11 muestras distintas. Se encontraron cristales en 36 muestras, en 20 de ellas de oxalato cálcico, en 11 de fosfato triple amónico magnésico y 5 de ellas de ácido úrico. Únicamente se identificó contaminación espermática en una muestra.

## Discusión

Aunque el análisis de orina es principalmente una prueba de detección, ofrece el perfil de varios parámetros que brindan una gran cantidad de información sobre posibles patologías renales y/o del tracto urinario, así como sobre patologías sistémicas [13]. Los niveles actuales de automatización y buena prestación analítica han cambiado el foco a la necesidad de optimizar la fase preanalítica para obtener resultados precisos. No siempre se logran las condiciones óptimas en la obtención de muestras, especialmente en los servicios de urgencias [14], lo que predispone a falsa positividad o negatividad de los test realizados.

La evaluación de interferencias en el recuento de eritrocitos mediante citofluorimetría evidenció la necesidad de que un analista experimentado revise el resultado antes de comunicarlo en todos aquellos casos en los que el valor obtenido mediante por la tira reactiva no coincida con el recuento automático. Aproximadamente en el 7 % de las muestras examinadas se produjo una sobreestimación del recuento eritrocitario, resultando en un falso positivo en la mayoría de los casos.

También se realizó una comparación de la evaluación de la esterasa leucocitaria mediante química seca (tira reactiva) y del análisis citofluorimétrico de leucocituria, confirmando la buena precisión de la almohadilla reactiva, tanto en el punto de corte de positividad (sensibilidad del 88 % y especificidad del 89 %) como en puntos de corte más alto (donde tanto la sensibilidad como la especificidad fueron superiores al 90 %).

Por otro lado, la evaluación de nitritos mediante tira reactiva (como estimación de la presencia de bacterias en la orina) tuvo un rendimiento deficiente, con una sensibilidad de solo el 41 % en el punto de corte de positividad. Esta baja sensibilidad se podría explicar por el hecho de que no todas las bacterias que provocan infecciones urinarias son reductoras de nitratos. Dada la elevada especificidad (99 %), un resultado positivo en la almohadilla reactiva de nitritos indica la presencia de bacterias en la muestra con un elevado grado de certeza.

El análisis del sedimento por microscopía digital proporcionó información de interés, ya que permitió detectar elementos formes de relevancia clínica en la mayoría de las muestras (71 %), precisando así un comentario descriptivo en el informe final. Dado que no existen almohadillas reactivas para detectar estos elementos, no se puede emplear únicamente la tira reactiva, ya que, de la presencia de cristales claramente patológicos, como los cristales de fosfato triple amónico magnésico, que se suelen encontrar en casos de infección del tracto genito-urinario, o para identificar una posible cristaluria en pacientes con sospecha de litiasis renal. Son necesarios más estudios para analizar dichos casos.

La evaluación y comunicación de los componentes morfológicos en el sedimento urinario, incluso en los servicios de urgencias, no solo mejora el rendimiento global de los análisis de orina, sino que proporciona información complementaria potencialmente importante. Huelga decir que la petición debe estar justificada y que la fase preanalítica debe ser rigurosa, con el fin de obtener muestras que ofrezcan información clínica valiosa.

## Supplementary Material

Supplementary Material

## Material Suplementario

Este artículo incluye material suplementario (https://doi.org/10.1515/almed-2024-0181).
