# Supplementary material for: Estudio sobre el valor incremental que supone el informe del sedimento en orina en medicina de urgencias mediante el sistema Sysmex® Serie-UN
Source: Adv Lab Med. 2024 Nov 26;5(4):381–5. [Article in Spanish] doi: 10.1515/almed-2024-0181 (PMC11661537; doi:10.1515/almed-2024-0181)
Supplement: Supplementary file 1 — Supplementary Material [file j_almed-2024-0181_suppl_001.docx]

**Material suplementario**

**Tabla 1:** Normas adoptadas por nuestro laboratorio sobre el uso del sistema UN de Sysmex (DMS-ANUR) para emplear la microscopía digital automática.

| **Norma** |
| --- |
| Búsqueda de cristales de fosfato triple amonico magnésico: pH > 8 + Bacterias > 1000 + Cilindros> 1 o Cristales > 10 |
| Espermatozoides ≥50 + Proteínas ≥15 |
| Cristales ≥30 |
| Cristales ≥15 + pH 5.0 o 5.5 o 6.0 |
| Células atípicas ≥5 |
| Células epiteliales no escamosas ≥10 |
| TranEC or RTEC ≥8 células |
| Cilindros hialinos ≥3 |
| Cilindros hialinos ≥2 + presencia de proteínas |
| Cilindros patológicos ≥1,5 |
| Cilindros patológicos ≥1,0 + presencia de proteínas |
| Levaduras ≥30 |
| Glucosa 500 o 1000 + Levaduras 10 - 30 |
| Aspecto turbio + sedimento negativo |
| Esterasa negativa + Leucocitos ≥150 |
| Leucocitos < 20 + Esterasa 2+ o 3+ |

**Tabla 2**: Listado de parámetros recogidos en el estudio, intervalo de referencia y estado actual del informe.

| **Parámetro** | **Unidades de medida** | **Intervalo de referencia** | **Informe de resultados** |
| --- | --- | --- | --- |
| **Análisis químico (químico y físico)** | | | |
| Hemoglobina | mg/dL | <0,03 | Se informa en analíticas ordinarias y urgentes |
| Esterasa leucocitaria |  | Ausente | Se informa en urgencias |
| Nitritos |  | Ausente | Se informa en analíticas ordinarias y urgentes |
| Glucosa | mg/dL | <10 | Se informa en analíticas ordinarias y urgentes |
| Cetonas | mg/dL | <5 | Se informa en analíticas ordinarias y urgentes |
| Bilirrubina | mg/dL | <0,2 | No se informa |
| Urobilinógeno | mg/dL | <0,5 | No se informa |
| Creatinina | mg/dL | 20-300 | No se informa |
| Proteína | mg/dL | <15 | Se informa en analíticas ordinarias y urgentes |
| Relación Proteína/Creatinina | mg/gCr | <150 | Se informa en analíticas ordinarias y urgentes |
| Albúmina | mg/L | <20 | No se informa |
| Relación Albúmina/Creatinina | mg/gCr | <30 | Se informa en analíticas ordinarias y urgentes |
| pH |  | 5,5-7,5 | Se informa en analíticas ordinarias y urgentes |
| Densidad relativa (peso específico) |  | 1,005-1,030 | Se informa en analíticas ordinarias y urgentes |
| Color |  | Amarillo | Se informa en analíticas ordinarias y urgentes (sólo si es anormal) |
| Apariencia |  | Limpio | No se informa |
| **Examen morfológico (Sedimento)** | | | |
| Eritrocitos | n°/ µL | 0-15 | Se informa en analíticas ordinarias |
| Leucocitos | n°/ µL | 0-20 | Se informa en analíticas ordinarias |
| Bacterias | n°/ µL | Ausente | Se informa en analíticas ordinarias |
| Células escamosas | n°/ µL | 0-20 | Se informa en analíticas ordinarias |
| Células no escamosas | n°/ µL | 0-8 | No se informa |
| Células atípicas | n°/ µL | 0-5 | No se informa |
| Cilindros de hialino | n°/ µL | 0-2 | No se informa |
| Cilindros patológicos | n°/ µL | 0-1 | No se informa |
| Cristales | n°/ µL | 0-15 | No se informa |
| Levaduras | n°/ µL | 0-30 | No se informa |
| Espermatozoides | n°/ µL | 0-50 | No se informa |
| Mucosidad | n°/ µL | 0-40 | No se informa |
| Conductividad | mS/cm | 3-38 | Se informa en analíticas ordinarias y urgentes |
